# THE RELATIONSHIP BETWEEN SUBJECTIVE AGE, CHRONOLOGICAL AGE, AND COGNITIVE FUNCTIONING IN OLDER ADULTS.

**DOI:** 10.1093/geroni/igz038.3278

**Published:** 2019-11-08

**Authors:** Emily P Morris, Laura B Zahodne

**Affiliations:** University of Michigan, Ann Arbor, Michigan, United States

## Abstract

Prior longitudinal research suggests that younger subjective age (i.e., feeling younger than your chronological age) predicts better subsequent cognitive functioning and lower dementia incidence independent of chronological age. However, no research has investigated interactions between subjective age and chronological age. This study examined whether older adults with more youthful subjective age performed better on cognitive evaluations than those with older subjective age and whether associations differed as a function of chronological age. Data from 1,047 older adults aged 65 and older from the Health and Retirement Study’s 2016 Harmonized Cognitive Aging Project were analyzed. Separate linear regressions estimated associations between subjective age and factor scores corresponding to five cognitive domains: executive function, episodic memory, language, visuospatial functioning, and processing speed. Covariates included sociodemographic characteristics and chronic disease burden. Interaction terms tested whether chronological age modified associations between subjective age and cognition. In the whole sample, younger subjective age was associated with better language. Significant interactions for all five domains revealed that associations were stronger and statistically significant for participants at the oldest chronological ages. The predictive value of subjective age may be highest among the oldest adults. These cross-sectional findings suggest that the oldest adults are most vulnerable to the detrimental effects of feeling older than one’s chronological age, cognitive difficulties are more relevant for subjective age perceptions for the oldest adults, and/or subjective age better reflects consequential physiological deterioration in that group. Future longitudinal research is needed to elucidate these possibilities.

